# Clinical and immunological outcomes of SARS-CoV-2-infected vaccine responders, vaccine non-responders, and unvaccinated patients evaluated for neutralizing monoclonal antibody treatment at a single German tertiary care center: a retrospective cohort study with prospective follow-up

**DOI:** 10.1007/s15010-023-02171-z

**Published:** 2024-02-02

**Authors:** J. Triebelhorn, J. Schneider, C. D. Spinner, R. Iakoubov, F. Voit, L. Wagner, J. Erber, K. Rothe, A. Berthele, V. Pernpeintner, E.-M. Strauß, L. Renders, A. Willmann, M. Minic, E. Vogel, C. Christa, D. Hoffmann, U. Protzer, S. D. Jeske

**Affiliations:** 1 Department of Internal Medicine II, University Hospital rechts der Isar, Technical University of Munich, Ismaninger Straße 22, 81675 Munich, Germany; 2 Department of Neurology, University Hospital rechts der Isar, Technical University of Munich, Munich, Germany; 3 Department of Nephrology, University Hospital rechts der Isar, Technical University of Munich, Munich, Germany; 4 Institute of Medical Microbiology, Immunology and Hygiene, School of Medicine, Technical University of Munich, Munich, Germany; 5 Institute of Virology, School of Medicine, Technical University of Munich/Helmholtz Centre Munich, Munich, Germany

**Keywords:** COVID-19, SARS-CoV-2, nMABs, Monoclonal antibodies, Immunosuppression, B-cell depletion

## Abstract

**Purpose:**

This study assessed the clinical and immunological outcomes of SARS-CoV-2-infected patients with risk factors for severe disease depending on their immunological status.

**Methods:**

In this retrospective study with single follow-up visit, clinical outcome and humoral immunity was monitored in SARS-CoV-2 infected patients at risk. The results were compared based on the patients’ initial immunological status: unvaccinated (UV), patients who did not develop neutralizing antibodies after vaccination (vaccine non-responders, VNR), and patients who expressed neutralizing antibodies after vaccination (vaccine responders, VR). Patients who lacked neutralizing antibodies (VNR and UV) were treated with nMABs.

**Results:**

In total, 113 patients at risk of severe COVID-19 consented to participate in the study. VR and UV were not admitted to the hospital. During the observation period, UVs had the highest rate of SARS-CoV-2 re-infections. Three of 41 VNRs (7.3%) were hospitalized due to severe COVID-19, with two of them having undergone iatrogenic B-cell depletion. The humoral immune response after infection was significantly lower in the VNR group than in the VR group in terms of anti-N, anti-receptor-binding domain (RBD), anti-S antibody titers, and anti-S antibody avidity. In a sub-analysis of VNR, B cell-deficient non-responders had significantly lower levels of anti-N antibodies and anti-S avidity after infection than other VNRs.

**Conclusion:**

VNR, particularly B-cell-depleted VNR, remained at risk of hospitalization due to COVID-19. In the VR group, however, no clinical complications or severe disease were observed, despite not receiving nMAbs. Tailoring the administration of nMABs according to patient vaccination and immunological status may be advisable.

**Supplementary Information:**

The online version contains supplementary material available at 10.1007/s15010-023-02171-z.

## Introduction

Neutralizing monoclonal antibodies (nMABs) targeting the novel severe acute respiratory coronavirus 2 (SARS-CoV-2) are an effective and specific treatment option during early infection for patients at high risk of disease progression towards severe coronavirus disease 2019 (COVID-19). nMABs are derived from a single B-cell clone and only target a single viral epitope. In the case of SARS-CoV-2, the current monoclonal antibodies mostly target the receptor-binding domain (RBD), a spike glycoprotein subunit essential for viral entry [1]. By binding to this site, nMABs inhibit its entry, thereby neutralizing SARS-CoV-2 and preventing disease progression. Additionally, virus inactivation triggered by conformational changes in the spike protein via nMABs has also been reported [2]. Multiple clinical trials have shown beneficial results in high-risk individuals in terms of mortality, prevention of hospitalization, and development of severe COVID-19. Casirivimab/Imdevimab and Sotrovimab both show a reduced risk of hospitalization and death against the ancestral strain as well as Alpha, Beta, and Delta variants [3]. However, because of the high plasticity of the RBD, SARS-CoV-2 variants can escape nMABs through mutations, thereby evading neutralization [4, 5]. This became obvious when the Omicron variant arose with prior nMAB treatments using casirivimab/imdevimab losing its efficacy. Since registrational trials of monoclonal antibodies have been carried out in seronegative and unvaccinated patients, the benefits of nMAB treatment in vaccinated, convalescent, or patients with hybrid immunity still need to be fully understood [6, 7].

This retrospective study aimed to assess the clinical and immunological outcomes of SARS-CoV-2 infected patients with risk factors for severe disease depending on their vaccine response status.

## Methods

### Ethics

The study was conducted following the principles of the Declaration of Helsinki. This study was approved by the Ethics Committee of the Technical University of Munich (approval no. 2022-216-S-SR). All patients with SARS-CoV-2 infection evaluated for nMABs therapy at our center were assessed for the study. All participants enrolled in this prospective follow-up study provided written informed consent.

### Clinical practice and recruitment

A total of 272 SARS-CoV-2-infected patients with relevant risk factors for disease progression towards (severe) COVID-19 were assessed for nMAB treatment in routine clinical care at our center. A total of 113 patients with SARS-CoV-2 infections between the 11th of November 2021 and the 31th of March 2022 agreed to participate in this study. Risk factors included age > 65 years, obesity (BMI > 30 kg/m^2^), cardiovascular disease, chronic lung disease, diabetes mellitus, chronic kidney disease, chronic liver disease, and immunosuppression (oncological disorder, organ or bone marrow transplant, human immunodeficiency virus (HIV) infection and immunosuppressive medication, as listed in Table 2). The indication for nMAB treatment was defined as follows: Individuals with SARS-CoV-2 infection at risk of severe COVID-19 who lacked anti-S neutralizing antibodies (NAB) in serological assessment before nMAB treatment due to either a lack of vaccination (unvaccinated, UV) or a failed humoral immune response following immunization (vaccine non-responder, VNR). In contrast, patients who tested positive for NAB against SARS-CoV-2 in the serological assessment did not undergo nMAB treatment, regardless of the NAB titer. Patients were closely monitored for one hour following intravenous nMAB treatment in our unit for side effects or reactions. Because of the immune-evading properties of the surging Omicron variant, the previously used nMABs casirivimab/imdevimab had to be replaced by sotrovimab in January 2022. Latest neutralizing data showed that casivirimab/imdevimab proved to be largely inactive against Omicron BA.1 and BA.2, while sotrovimab still remained neutralizing activity [8]. All the patients evaluated for nMAB treatment were invited to participate in the study. The participants were assessed for their premedical history, COVID-19 vaccination status, course of SARS-CoV-2 infection, and reinfection. If the patients agreed to participate, serological blood samples and SARS-CoV-2 swabs were obtained during a single follow-up visit.

### Humoral immunity

SARS-CoV-2 anti-N antibodies and NAB in participants’ sera were measured using the iFlash 1800 platform (YHLO Biotechnology, Shenzhen, China). The iFlash-SARS-CoV-2 IgG antibody CLIA was performed following the manufacturer’s instructions for anti-N antibodies. For NAB, we used the iFlash 2019-nCoV NAb CLIA, based on the competition between serum antibodies with recombinant angiotensin-converting enzyme 2 to bind to the SARS-CoV-2 Wuhan strain receptor-binding domain. According to the manufacturer's instructions, this surrogate neutralization assay was adapted for quantification, and the cutoff level for seropositivity was set at 10 neutralizing units per ml (AU/ml). The maximum measurable NAB value was 800 AU/ml.

To assess the participants’ SARS-CoV-2 anti-S antibodies, the Abbott SARS-CoV-2 IgG II Quant immunoassay was performed on the Abbott Architect i1000SR platform (Abbott, Illinois, USA) according to the manufacturer’s instructions. The analytical measurement range is 21 to 40,000 AU/ml and the cutoff for seropositivity is ≥ 50 AU/ml. The binding strength of anti-S-IgG avidity was determined by the adaptation of IgG agile SARS-CoV-2 ELISA (Virion/Serion, Würzburg, Germany) using ammonium thiocyanate (NH4SCN, Roth, Karlsruhe, Germany) as a chaotropic agent, as described previously. Briefly, serum samples were adjusted to 100 IU/mL antibody activity, according to the standard curve provided by the manufacturer, to exclude the influence of different IgG concentrations. The samples were then incubated in pre-coated plates with SARS-CoV-2 spike ectodomain antigens (Virion/Serion) for 1 h at 37 °C in a humid chamber. After washing steps, antigen–antibody complexes were incubated in the presence of 1.0 M NH_4_SCN or phosphate-buffered saline (PBS, control) for 10 min at room temperature. After washing steps, a detection antibody was added, and ELISA was performed according to the manufacturer’s instructions. The relative avidity index (RAI) was calculated as the ratio of IgG concentration (NH_4_SCN) to IgG concentration (PBS). Values were interpreted as low (< 40%), moderate (40% to 60%), and high RAI (> 60%) analogous to experiences with other viral antigens.

### Statistics

Based on vaccination status and NAB titers in sera at the initial presentation, clinical and serological results were compared between three groups: unvaccinated (UV, received no vaccinations), vaccine responders (VR, expressed ≥ 10 AU/ml NAB at initial visit following at least one vaccination), and vaccine non-responders (VNR, expressed < 10 AU/ml NAB at initial visit following at least one vaccination). Scatterplots are displayed with mean ± standard error, and groups were compared using Kruskal–Wallis or Mann–Whitney *U* tests. At *p* ≤ 0.05, the differences were considered significant. GraphPad Prism Version 10.0.2 by Graphpad Prism Inc., San Diego, California, USA, was used to calculate and plot the results.

## Results

### Baseline characteristics

**Fig. 1:**
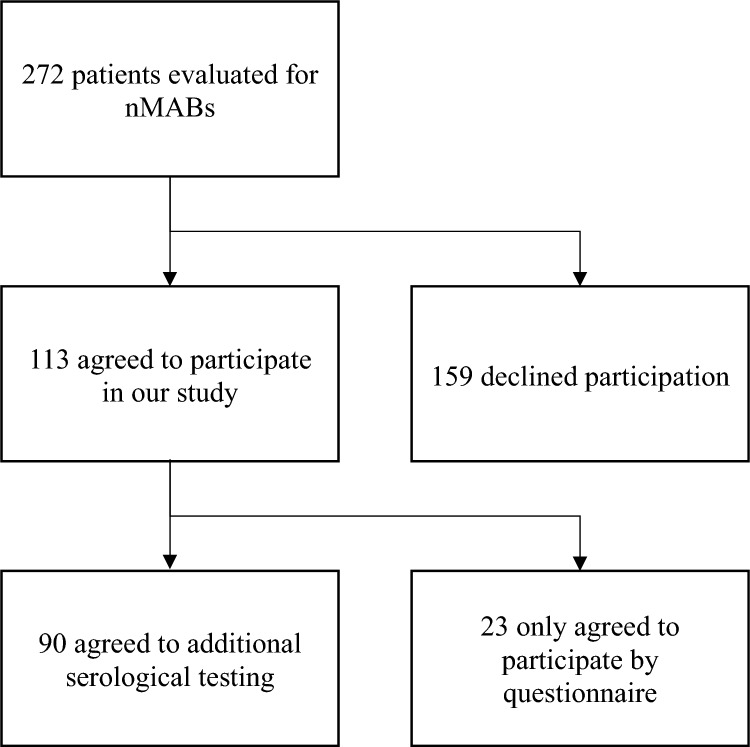
Flowchart depicting included patients

In total, 227 patients were evaluated for nMAB treatment; 113 consented to participate in this study and 90 agreed to additional serological testing (Fig. 1).

The median age was 59 (QR 26–88) years. 57 patients were female and 56 were male. Twenty-five individuals were unvaccinated, whereas 88 received at least one vaccination before presentation. In the vaccinated group, 41 individuals demonstrated no humoral response and were classified as VNR. Thirty-five patients were infected with the Delta variant and 78 with the Omicron BA.1/BA.2 variant. Among our participants, nMABs were administered to a total of 66/113 (58.4%) individuals. Among them, 26/66 (39.4%) patients received casirivimab/imdevimab, and 40/66 (60.1%) received sotrovimab. The distribution of comorbidities and immunosuppressive medication is shown in Table 1, 2. In Table 3, we explore the distribution of VNRs over the main risk factors for vaccine non-responsiveness in our cohort: oncological disease, organ transplant and B-cell depletion (iatrogenically, either due to multiple sclerosis or rheumatologial disease).

**Table 1:** Distribution of prominent comorbidities in participating patients

COPD	Asthma	Arterial hypertension	CHD	Arrhythmia	CKD	Dialysis	Diabetes	Autoimmune liver disease	IBD	MS	Organ transplant	Oncologial disease	HIV
5	14	30	8	12	12	2	8	3	2	19	11	30	1

*COPD* Chronic obstructive pulmonary disease, *CHD* coronary heart disease, CKD chronic kidney disease, *IBD* inflammatory bowel disease, *MS* multiple sclerosis, *HIV* human immunodeficiency virus

**Table 2:** Distribution of immunosuppressive medications

Ocrelizumab	Fingolimod	Corticosteroids	Tacrolimus	MMF	MTX	Rituximab	Cyclosporine	Everolimus	Pembrolizumab	Chemotherapy	Checkpoint-inhibitor
10	5	10	8	3	1	6	2	1	1	20	2

*MMF* Mycophenolate mofetil, *MTX* methotrexate

**Table 3:** Main comorbidities of VNR

	Oncologial disease	Organ transplant	B-cell depletion	None oft the other
Total	30	11	16	56
VNR	17 (56.7%)	6 (54.5%)	14 (87.5%)	4 (7.1%)

Ratio of VNR with comorbidity/patients with comorbidity in our cohort

### Clinical outcome

No deaths occurred during the follow-up period (median, 158 (QR 103–237) days). Flu-like symptoms such as fever, coughing, sore throat, headache, as well as abdominal pain, diarrhea, nausea, and loss of taste and smell were experienced by patients in all three groups. There were no significant differences in the occurrence of symptoms between groups. The duration of symptoms also did not differ significantly between groups (median, 7 (QR 4–12) days). No hospitalizations were reported for VR and UV, whereas three VNRs (7.3%) required hospitalization due to severe COVID-19. Before hospital admission, all three patients received nMABs because of their lack of NAB despite being vaccinated. Our hospitalized patients included a 62-year-old female patient who received rituximab for multiple sclerosis, a 68-year-old male patient with B-cell chronic lymphatic leukemia who was treated with obinutuzumab, and a 77-year-old male patient who also had B-cell chronic lymphatic leukemia but did not receive any therapy. nMABs were administered 2, 5, and 30 days after the initial diagnosis. The patient who received sotrovimab 30 days after the initial diagnosis had progressive respiratory symptoms and a persistent viral load in nasopharyngeal swabs. Four days after therapy, he then fully cleared SARS-CoV-2 and was discharged in an improved condition. None of the hospitalized patients were required to be transferred to an intensive care unit. During the follow-up period, reinfections were the highest in UV individuals (20%), followed by VNRs (14.6%), whereas only 2.1% of theVRs experienced reinfection. The mortality, hospitalization, and reinfection rates for the VR, VNR, and UV groups are shown in Table 4.

**Table 4:** Comparison of hospitalization and reinfection between VNR, VR and UV

	Total	Hospitalized because of COVID-19		Reinfected with SARS-CoV-2	
		Total	Percentage	Total	Percentage
VNR	41	3	7.3	6	14.6
VR	47	0	0	1	2.1
UV	25	0	0	5	20
Total	113	3		13	

### Humoral immune response after presentation in our outpatient nMAB center

**Fig. 2:**
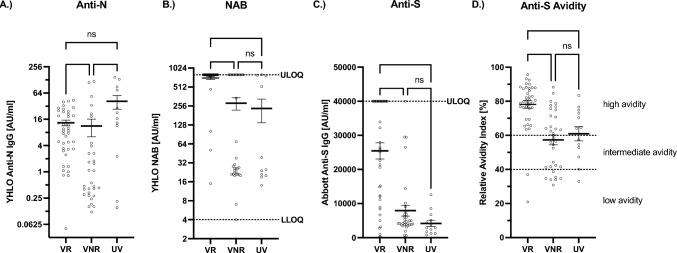
Comparison of humoral immunity following infection between UV, VNR, and VR. **A**–**D** Patient sera were analyzed for NAB, anti-N- and anti-S-antibodies as well as anti-S-antibody avidity. Bar and whiskers mark the mean with standard error. Sera of UV (*n* = 13), VNR (*n* = 34), and VR (*n* = 40) were compared. Statistical analysis was performed via the Kruskal–Wallis test with Dunn's correction. ****p < 0.0001, ***p < 0.001, **p < 0.01, n.s. not significant. **A** VR (mean = 13; SEM =  ± 2); VNR (mean = 11; SEM =  ± 5); UV (mean = 42; SEM =  ± 14); VR vs. VNR (*p* = 0.0005); VR vs. UV (*p* =  > 0.999); VNR vs. UV (*p* = 0.0086). **B** VR (mean = 711; SEM =  ± 37); VNR (mean = 283; SEM =  ± 63); UV (mean = 234; SEM =  ± 95); VR vs. VNR (*p* =  < 0.0001); VR vs. UV (*p* =  < 0.0001); VNR vs. UV (*p* = 0.64). **C** VR (mean = 25,446; SEM =  ± 2370); VNR (mean = 7930; SEM =  ± 1588); UV (mean = 4180; SEM =  ± 940); VR vs. VNR (*p* =  < 0.0001); VR vs. UV (*p* =  < 0.0001); VNR vs. UV (*p* = 0.719). **D** VR (mean = 78; SEM =  ± 2); VNR (mean = 42; SEM =  ± 3); UV (mean = 61; SEM =  ± 4); VR vs. VNR (*p* =  < 0.0001); VR vs. UV (*p* = 0.0016); VNR vs. UV (*p* =  > 0.999)

**Fig. 3:**
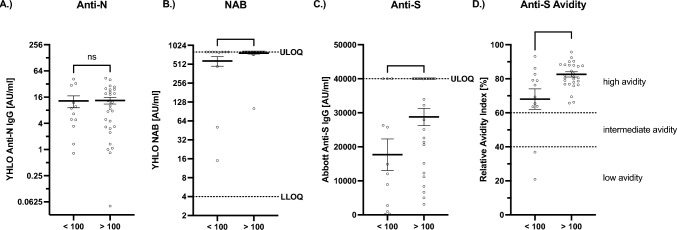
Subanalysis of VR, comparison of humoral immunity following infection in dependence of initial nAB titer. **A**–**D** VR sera were analyzed for NAB, anti-N- and anti-S-antibodies as well as anti-S-antibody avidity. Bar and whiskers mark the mean with standard error. Sera of VR with an initial titer below 100 AU/ml (*n* = 12) and above 100 AU/ml (*n* = 28) were compared. Statistical analysis was performed via Mann–Whitney *U* test. *p < 0.05, n.s. not significant. **A** < 100 (mean = 13.1; SEM =  ± 4.0); > 100 (mean = 13.4; SEM =  ± 2.3); *p* = 0.9936; two-tailed. **B** < 100 (mean = 576.8; SEM =  ± 100.3); > 100 (mean = 768.8; SEM =  ± 25.0); *p* = 0.048; two-tailed. **C** < 100 (mean = 17,682.0; SEM =  ± 4657.0); > 100 (mean = 28,773.0; SEM =  ± 2537.0); *p* = 0.042; two-tailed. **D** < 100 (mean = 68.1; SEM =  ± 6.0); > 100 (mean = 78.7; SEM =  ± 1.6); *p* = 0.011; two-tailed

**Fig. 4:**
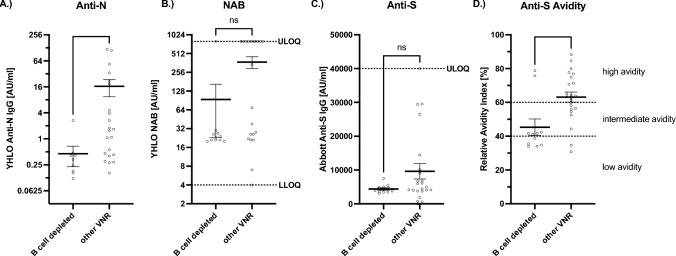
Subanalysis of B-Cell depleted patients, comparison of humoral and cellular immunity following infection between B-cell depleted patients and other VNR. **A**–**D** VNR sera were analyzed for NAB, anti-N- and anti-S-antibodies as well as anti-S-antibody avidity. Bar and whiskers mark the mean with standard error. Sera of VNRs undergoing B cell depletion (*n* = 11) and and non-B-cell-depleted VNRs (*n* = 23) were compared. Statistical analysis was performed via Mann–Whitney *U* test. **p < 0.01, n.s. not significant. **A** B cell depleted (mean = 0.45; SEM =  ± 0.23); other VNR (mean = 16.45; SEM =  ± 7.04); *p* = 0.0025; two-tailed. **B** B cell depleted (mean = 93.9; SEM =  ± 70.6), other VNR (mean = 373.9; SEM =  ± 80.8); *p* = 0.1027; two-tailed. **C** B-cell depleted (mean = 4390; SEM =  ± 381.3); other VNR (mean = 9624; SEM =  ± 2271); *p* = 0.1643; two-tailed. **D** B cell depleted (mean = 45.3; SEM =  ± 4.9); other VNR (mean = 63.1; SEM =  ± 3.0); *p* = 0.0066; two-tailed

Both UV and VNR demonstrated significantly lower levels of NAB and anti-S antibodies as well as anti-S antibody avidity compared to VR. In contrast, anti-N antibodies were only significantly lower in VNR than in the other groups (Fig. 2A–D). A sub-analysis of VR with NAB < 100 AU/ml (subgroup 1) and NAB > 100 AU/ml (subgroup 2) at first presentation in our outpatient clinic for COVID-19 nMABs treatment revealed significantly lower NAB and anti-S antibodies, as well as anti-S avidity in patients who initially presented with anti-S < 100 AU/ml (Fig. 3B–D). Anti-N antibody responses were not significantly different (Fig. 3A). We also compared the humoral immune response after SARS-CoV-2 infection in B cell-depleted patients (*n* = 16) with that in VNR patients (*n* = 23) who did not receive B cell-depleting medication. All the patients included in this sub-analysis received nMABs. Compared to (other) VNR, B cell-depleted patients expressed significantly lower titers of anti-N antibodies after infection while generating similar NAB and anti-S antibody responses with significantly lower anti-S antibody avidity (Fig. 4A–D).

## Discussion

In our German tertiary care center, SARS-CoV-2-infected patients with risk factors for severe COVID-19 were evaluated for nMAB treatment. Following our in-house approach, the administration of nMABs was carried out with strict criteria. In general, nMABs were restricted to patients with an early SARS-CoV-2 infection (≤ 5 days after symptom onset or diagnosis) who were at risk of developing severe COVID-19 and showed no detectable NAB due to either immunosuppression or a lack of vaccination. In this retrospective cohort analysis, we compared the clinical and serological outcomes of the VR, VNR, and UV groups. Hospitalization occurred solely in VNRs (7.3%), who received nMABs before hospital admission. In our serological characterizations after infection, the levels of NAB, anti-S and anti-N antibody titers, as well as anti-S antibody avidity, were significantly lower in VNR than in VR, pointing to a persisting inability to generate an adequate humoral immune response if infected. The VNR group is predominantly comprised of individuals with high levels of immunosuppression, either due to underlying medical conditions (oncological disease) or immunosuppressive medications (B-cell depletion, organ transplant). Of the 41 VNR, 17 have an oncological disease, 6 are organ transplanted, and 14 patients are iatrogenically B-cell depleted either due to multiple sclerosis or rheumatological disease (Table 3). Looking at the likelihood of vaccine non-responsiveness in regard to these comorbidities, B-cell-depleted patients had by far the highest chance of being VNR, with 87,5% of B-cell-depleted patients lacking a humoral immune response after vaccination. The percentage of VNR in oncological and organ transplanted patients was also notable with 56,7% and 54,5%, making these three comorbidities the main risk factors for an impaired humoral immune response either after vaccination or infection in our cohort. Taking a closer look at the hospitalized VNR, all three patients had impaired B-cell function: two had received B-cell-depleting medications, and the other suffered from B-cell lymphatic leukemia. Consistent with our clinical findings, iatrogenically B cell-depleted patients also expressed significantly lower levels of anti-N antibodies and reduced avidity of anti-S antibodies than non-B cell-depleted VNR after infection. These findings underscore the impaired capacity of B cell-depleted patients to generate an adequate humoral immune response, even when compared to other immunosuppressed individuals.

In contrast, VR, irrespective of their NAB titers, avoided hospitalization without receiving nMABs and exhibited the lowest rates of reinfections throughout the follow-up period. In addition to the clinical outcomes, our serological analysis showed that patients with low NAB titers (< 100 AU/ml) at the time of the initial presentation had significantly lower levels of NAB and anti-S antibodies at the follow-up visit. Additionally, they exhibited a significantly reduced avidity of anti-S antibodies following infection compared to patients with high NAB titers (> 100 AU/ml). These findings suggest a relationship between antibody titers and avidity before and after infection. Individuals exhibiting diminished antibody titers post-vaccination, likely attributed to immunosuppression, also appear to develop lower humoral immunity following a natural infection. However, our limited clinical data suggest no difference in susceptibility between patients with low (< 100 AU/ml) NAB titers and high NAB titers.

Hospitalization was not observed in the UV group, although this group had the highest rate of reinfections. Most UV strongly opposed vaccination and persisted in their refusal to be vaccinated after receiving the nMABs, which could explain the high reinfection rate observed in this group. The UV group showed a significantly lower SARS-CoV-2 specific humoral response regarding NAB and anti-S titers, as well as anti-S avidity than the VR group. These findings align with our previous study, highlighting the role of infection plus vaccination-induced hybrid immunity in generating high-quality humoral responses [9].

The limitations of this study include a heterogeneous sample and varying time periods between infection and re-evaluation. Our patients suffered from a wide range of illnesses and were immunocompromised. We evaluated this heterogeneous population by focusing on humoral immunization status. Comparing the rates of VNR in oncological patients, organ transplanted patients and B-cell depleted patients to literature, our proportion of VNR is distinctly higher [10]. However, our cohort has undergone a possible selection bias. Treating physicians tended to send their more immunocompromised patients to be evaluated for nMabs therapy, naturally selecting towards patients more likely to lack a humoral immune response after vaccination. The time between the original evaluation, infection, and the subsequent re-evaluation ranged between 103 and 237 days, which may have affected the clinical and serological data, such as the number of reinfections and antibody titers. Additionally, when interpreting reinfections, it is vital to note that six reinfected patients were initially infected with the Delta variant and received casirivimab/imdevimab. Due to the timely manner in which the reinfection occurred, these patients were likely reinfected with the omicron variant, which has been proven to evade prior immune responses. The serological measurement of antibodies against SARS-CoV-2 is widely utilized in clinical routine to assess the immune response following infection or vaccination. However, the commercially available assays also used in this study were developed based on the SARS-CoV-2 Wuhan strain. Due to antigenic shifts, the antibody titers of patients following SARS-CoV-2 infection with Delta or Omicron variants may be higher than detected [11]. Although NAB titers are generally recognized as highly predictive for immune protection against symptomatic SARS-CoV-2 infection, this highlights that there is a need to update the commercial assays commonly used for SARS-CoV-2 antibody detection [12–14]. Measurement of NAB and anti-S antibody titers and anti-S antibody avidity may have also been impaired by nMAB treatment since discrimination between circulating nMABs and endogenous antibodies is not possible. When calculating anti-S antibody titers in relation to the time elapsed since infection, there was no relevant decline in antibodies, as expected, if monoclonal antibodies were still present at the time of measurement (Appendix, Fig. 1). Therefore, it is unlikely that the application of nMABs influenced the observed humoral response.

To summarize our observations, VR with risk factors for severe COVID-19 managed SARS-CoV-2 infection without hospitalization despite not receiving nMABs and showed a robust humoral response after infection. In contrast, VNRs who received nMABs still had severe COVID-19 and needed to be hospitalized in three cases (7.3%). The humoral immune responses following infection were also significantly lower in the VNR group, highlighting its persistent susceptibility, with B-cell-depleted patients being especially vulnerable. Our findings suggest that tailoring the administration of nMABs according to the patients’ vaccination and immunological status may be advisable.

### Supplementary Information

Below is the link to the electronic supplementary material.

Supplementary file1 (DOCX 83 KB)

## Data Availability

The participants of this study did not give written consent for their data to be shared publicly, so due to the sensitive nature of the research supporting data is not available.

## Abbreviations

nMABs: Neutralizing monoclonal antibodies
RBD: Receptor binding domain
NAB: Neutralizing antibodies
VR: Vaccine-responder
VNR: Vaccine non-responder
UV: Unvaccinated
