# Supplementary material for: Clinical and immunological outcomes of SARS-CoV-2-infected vaccine responders, vaccine non-responders, and unvaccinated patients evaluated for neutralizing monoclonal antibody treatment at a single German tertiary care center: a retrospective cohort study with prospective follow-up
Source: Infection. 2024 Feb 2;52(3):1143–51. doi: 10.1007/s15010-023-02171-z (PMC11143028; doi:10.1007/s15010-023-02171-z)
Supplement: Supplementary file 1 — Supplementary file1 (DOCX 83 KB) [file 15010_2023_2171_MOESM1_ESM.docx]

**Clinical and immunological outcomes of SARS-CoV-2-infected vaccine responders, vaccine non-responders, and unvaccinated patients evaluated for neutralizing monoclonal antibody treatment at a single German tertiary care center: A retrospective cohort study with prospective follow-up**

J. Triebelhorn^1^, J. Schneider^1^ , C. D. Spinner^1^ , R. Iakoubov^1^ , F. Voit^1^ , L. Wagner^1^ , J. Erber^1^, K. Rothe^4^, A. Berthele^2^ , V. Pernpeintner^2^, E.-M. Strauß^2^ , L. Renders^3^ , A. Willmann^5^ , M. Minic^5^ , E. Vogel^5^ , C. Christa^5^, D. Hoffmann^5^, U. Protzer^5^ and S. D. Jeske^5^

1 University hospital rechts der isar, Department of Internal Medicine II, Technical University of Munich, Munich, Germany

2 University hospital rechts der isar, Department of Neurology, Technical University of Munich, Munich, Germany

3 University hospital rechts der isar, Department of Nephrology, Technical University of Munich, Munich, Germany

4 University hospital rechts der Isar, Institute for Medical Microbiology, Immunology and Hygiene, Technical University of Munich, Munich, Germany

5 Institute of Virology, School of Medicine, Technical University of Munich/Helmholtz Centre Munich, München, Deutschland

Corresponding Author: Julian Triebelhorn, Klinikum rechts der Isar, Ismaninger Straße 22, 81675 München, Germany, [julian.triebelhorn@mri.tum.de](mailto:julian.triebelhorn@mri.tum.de)

**Supplementary information**

Figure S1: Anti-S antibody titers according to time since infection
